# Letter to the Editor in Regards to “The Characteristics of Sinonasal Inverted Papilloma and Recurrence Factors: An Analysis of 207 Cases”

**DOI:** 10.1002/wjo2.70117

**Published:** 2026-08-24

**Authors:** Valli Rajasekaran, Preethi Ekambaram

**Affiliations:** ^1^ Department of Otorhinolaryngology, Shri Sathya Sai Medical College and Research Institute Sri Balaji Vidyapeeth (Deemed to be University) Puducherry India

## Author Contribution

Both authors contributed equally to the conception, drafting, and revision of the manuscript and approved the final version.

## Funding

The authors have nothing to report.

## Conflicts of Interest

The authors declare no conflicts of interest.

To the Editor,

We read with interest the retrospective analysis by SamimiArdestani et al. [1] on the clinicopathologic characteristics and recurrence patterns in 207 patients with sinonasal inverted papilloma. The authors' work deserves credit for presenting one of the largest single‐centre series in recent years. That said, a few points regarding the methodology and analysis would benefit from further clarification to help readers interpret the findings more accurately.

First, a recurrence rate of 26.6% was reported, but the author does not mention the follow‐up duration and the surveillance protocol used to detect recurrence. Adding the follow‐up time distribution and methods of detection ‐endoscopy, imaging, or clinical review would allow for meaningful comparison with other cohorts.

In addition, logistic regression is mentioned in the methods, but the results do not include multivariable results such as adjusted odds ratios, confidence intervals, and variables included in the model. This is essential for studying associations such as smoking and malignant transformation, which may be influenced by confounders like age or tumour site.

Finally, in this study, an MRI was obtained for 27 patients. Adding the criteria for choosing MRI will enhance the interpretation of conclusions.

We believe clarifying these aspects would strengthen the study and provide a better understanding of recurrence and malignancy risks in inverted papilloma.

## Data Availability

The data that support the findings of this study are available from the corresponding author upon reasonable request.
